# Severe Acute Respiratory Syndrome Coronavirus 2 P.2 Lineage Associated with Reinfection Case, Brazil, June–October 2020

**DOI:** 10.3201/eid2707.210401

**Published:** 2021-07

**Authors:** Paola Cristina Resende, João Felipe Bezerra, Romero Henrique Teixeira Vasconcelos, Ighor Arantes, Luciana Appolinario, Ana Carolina Mendonça, Anna Carolina Paixao, Ana Carolina Duarte, Thauane Silva, Alice Sampaio Rocha, Ana Beatriz Machado Lima, Alex Pauvolid-Corrêa, Fernando Couto Motta, Dalane Loudal Florentino Teixeira, Thiago Franco de Oliveira Carneiro, Francisco Paulo Freire Neto, Isabel Diniz Herbster, Anderson Brandao Leite, Irina Nastassja Riediger, Maria do Carmo Debur, Felipe Gomes Naveca, Walquiria Almeida, Mirian Livorati, Gonzalo Bello, Marilda M. Siqueira

**Affiliations:** Instituto Oswaldo Cruz, Rio de Janeiro, Brazil (P.C. Resende, I. Arantes, L. Appolinario, A.C. Mendonça, A.C. Paixao, A.C. Duarte, T. Silva, A.S. Rocha, A.B.M. Lima, A. Pauvolid-Corrêa, F.C. Motta, G. Bello, M.M. Siqueira);; Universidade Federal da Paraíba, João Pessoa, Brazil (J.F. Bezerra, R.H.T. Vasconcelos);; Texas A&M University, College Station, Texas, USA (A. Pauvolid-Corrêa);; Laboratório Central do Estado da Paraíba, João Pessoa (L.F. Teixeira, T.F. de Oliveira Carneiro);; Universidade Federal do Rio Grande do Norte, Natal, Brazil (F.P.F. Neto);; Maternidade Escola Januario Cicco, Natal (I.D. Herbster);; Laboratório Central do Estado do Alagoas, Maceió, Brazil (A.B. Leite);; Laboratório Central do Estado do Paraná, Curitiba, Brazil (I.N. Riediger, M.C. Debur);; Instituto Leônidas e Maria Deane, on behalf of the COVIDNORTE team, Manaus, Brazil (F.G. Naveca);; Ministério da Saúde do Brasil, Brasilia, Brazil (W. Almeida, M. Livorati)

**Keywords:** COVID-19, coronavirus disease, SARS-CoV-2, severe acute respiratory syndrome coronavirus 2, viruses, respiratory infections, zoonoses, reinfection, secondary infection, P.2, Brazil

## Abstract

A 37-year-old healthcare worker from the northeastern region of Brazil experienced 2 clinical episodes of coronavirus disease. Infection with severe acute respiratory syndrome coronavirus 2 was confirmed by reverse transcription PCR in samples collected 116 days apart. Whole-genome sequencing revealed that the 2 infections were caused by the most prevalent lineage in Brazil, B.1.1.33, and the emerging lineage P.2. The first infection occurred in June 2020; Bayesian analysis suggests reinfection at some point during September 14–October 11, 2020, a few days before the second episode of coronavirus disease. Of note, P.2 corresponds to an emergent viral lineage in Brazil that contains the mutation E484K in the spike protein. The P.2 lineage was initially detected in the state of Rio de Janeiro, and since then it has been found throughout the country. Our findings suggest not only a reinfection case but also geographic dissemination of the emerging Brazil clade P.2.

The efficiency and persistence of natural protective immunity caused by severe acute respiratory syndrome coronavirus 2 (SARS-CoV-2) infection or vaccination are currently unknown. Reinfection cases have been reported in different countries (*1*), but the differentiation between cases of reinfection and viral persistence remains a challenge. The detection of 2 coronavirus disease (COVID-19) episodes >90 days apart and caused by 2 different lineages of SARS-CoV-2 remains the most reliable evidence of reinfection (*2*). In this article, we describe a reinfection case and highlight details about the genomic features of the 2 COVID-19 episodes. In addition, we demonstrate that the virus in the second episode was related to the emerging variant of interest (VOI) designated as lineage P.2, which is currently circulating throughout Brazil.

## Methods

### Case Description

A 37-year-old female physician with no underlying conditions reported 2 episodes of COVID-19 in the state of Rio Grande do Norte in the northeastern region of Brazil. The first episode occurred in June 2020 and the second in October 2020; a total of 116 days occurred between the 2 episodes. 

On June 17, the case-patient, who lives in Rio Grande do Norte and works in the neighboring state of Paraíba, experienced symptoms such as headache, runny nose, diarrhea, and myalgia, and her illness was classified as a mild COVID-19 case with no complications (*3*). A nasopharyngeal swab specimen was collected on June 23, 6 days after the onset of symptoms. A second nasopharyngeal swab specimen was collected on September 16 as part of a follow-up procedure. On October 11, the patient experienced intense headache, ageusia, anosmia, and fatigue, which were suggestive of a new COVID-19 episode. This second infection was mild and also evolved without complications. On October 13, 2 days after the second onset of symptoms, a third nasopharyngeal swab specimen was collected.

### Ethics

This study was approved by the FIOCRUZ-IOC Ethics Committee (68118417.6.0000.5248 and CAAE 32333120.4.0000.5190) and the Ministry of Health of Brazil SISGEN (A1767C3). In addition, the case-patient read and signed the free and informed consent form.

### Procedures

First and third nasopharyngeal swab specimens were initially processed by the Public Health Central Laboratory of the state of Paraíba; the second nasopharyngeal swab specimen was processed by the Institute of Tropical Medicine of the Federal University of Rio Grande do Norte in northeastern Brazil. For the first and third specimens, viral RNA was extracted by using QIAamp Viral RNA Mini Kit (QIAGEN, https://www.qiagen.com), according to the manufacturer’s instructions. RNA samples were tested for SARS-CoV-2 by real-time reverse transcription PCR (rRT-PCR) using a molecular kit design for the targets envelope gene and internal control human RNase P gene (*4*). For the second nasopharyngeal swab specimen, we extracted RNA by using Extracta kit Viral DNA and RNA (MVXA-P016) (Loccus, https://loccus.com.br) and tested for SARS-CoV-2 by using a rRT-PCR probe for the targets N1, N2, and Rnase P (Integrated DNA Technologies, https://www.idtdna.com) (*5*). For confirmation and complementary analysis, positive samples were sent to the Laboratory of Respiratory Viruses and Measles at Fiocruz, Brazil’s National Reference Laboratory and the World Health Organization Reference Laboratory for Coronavirus.

According to the technical note of the Ministry of Health of Brazil 52/2020-CGPNI/DEIDT/SVS/MS, >2 rRT-PCR–positive swab samples collected >90 days apart, independent of clinical conditions, are required to confirm a SARS-CoV-2 reinfection. As the routine procedure for confirmation of reinfection cases, the 2 positive results obtained for this patient were confirmed by rRT-PCR. The RNA was obtained by using QIAamp Viral RNA Mini Kit (QIAGEN), according to the manufacturer’s instructions. Molecular detection of SARS-CoV-2 was performed by using a rRT-PCR Biomanguinhos SARS-CoV-2 kit for the targets E, N1, N2, and Rnase P (*4*,*5*) using the Applied Biosystems 7500 Real-Time PCR (Thermo Fisher Scientific, https://www.thermofisher.com).

For supplementary analysis, the nasopharyngeal swab specimens were submitted for the qualitative detection of SARS-COV-2 antigens by using the Panbio COVID-19 Ag rapid test device (Abbott, https://www.abbott.com), according to the manufacturer’s instructions. Both clinical samples were also submitted to virus isolation in cell cultures as previously described (*6*). Next, 200 μL of the viral transport medium of positive specimens were inoculated in VERO E6 cells flasks and inspected daily for cytopathic effect (CPE) in a total of two 4-day blind passages. SARS-CoV-2 CPE was confirmed by rRT-PCR of culture supernatant. In cases in which no CPE was observed, rRT-PCR was performed on day 4 to confirm absence of virus replication.

In addition, we tested the serum sample from the case-patient’s second episode of COVID-19 for IgG by the Abbott chemiluminescent microparticle immunoassay (CMIA) using nucleocapsid protein, as well as for SARS-CoV-2–specific neutralizing antibodies by plaque reduction neutralization test (PRNT) (*7*) for confirmation. For PRNT, an aliquot of serum sample inactivated at 56°C for 30 minutes was tested in VERO CCL-81 cells in duplicate at serial 2-fold dilutions to determine 90% endpoint titers against 4 infectious SARS-CoV-2 lineages, including B.1 (GISAID [https://www.gisaid.org] accession no. EPI_ISL_414045), P.1 (accession no. EPI_ISL_1402431), P.2 (accession no. EPI_ISL_1402429), and B.1.1.7 (accession no. EPI_ISL_1402430). Serum samples were considered seropositive when a serum dilution of at least 1:10 reduced >90% of the formation of SARS-CoV-2 viral plaques (PRNT_90_) (*7*).

We performed whole-genome sequencing by using the RNA extracted manually using the QIAamp Viral RNA Mini Kit (QIAGEN). The SARS-CoV-2 genomes were recovered by using Illumina or ONT Nanopore sequencing protocols previously established and used by the Fiocruz COVID-19 Genomic Surveillance Network to recover high-quality genomes (P.C. Resende, unpub. data, https://doi.org/10.1101/2020.04.30.069039). The FASTQ reads obtained were imported into the CLC Genomics Workbench version 20.0.4 (QIAGEN), trimmed, and mapped against the reference sequence EPI_ISL_402124 from GISAID. The alignment was refined by using the InDels and Structural Variants module, then the Local Realignment module and the final consensus obtained. Maximum-likelihood phylogenetic analysis of all SARS-CoV-2 whole genomes from the state of Paraíba was conducted by using PhyML (*8*). We conducted Bayesian phylogeographic analysis in BEAST version 1.10 (*9*).

## Results

### Diagnostic Laboratory Findings

The first and third nasopharyngeal swab specimens, collected on June 23 and October 13, 2020, tested positive for SARS-CoV-2 by rRT-PCR, whereas the second nasopharyngeal swab specimen, collected on September 8, tested negative. Both positive specimens had high viral load, presumed by the low cycle threshold (C_t_) values by rRT-PCR (Table). The 2 positive samples were confirmed by using the rRT-PCR protocol and the Ag-RDT Panbio COVID-19 Antigen Test (Abbott) directly from the clinical sample.

**Table:** Laboratory test results of severe acute respiratory syndrome coronavirus 2 reinfection case-patient, Brazil, June–October 2020*

Clinical specimens	Symptom onset	Collection date	RT-PCR SARS-CoV-2	RT-PCR SARS-CoV-2 (LVRS, Fiocruz)	Sequencing GISAID clade/PANGO lineage	Antigen test (Abbott)	IgG	Neutralization assay
NPS, sample 1	2020 Jun 17	2020 Jun 23	Positive, C_t_ E = 22, C_t_ RP = 21 (LACEN-PB)	Positive, C_t_ E = 24, C_t_ N1 = 24, C_t_ N2 = 25, C_t_ RP = 26	GR/B.1.1.33	Positive	NA	NA
NPS, sample 2	Asymptomatic	2020 Sep 8	ND, C_t_ E = ND, C_t_ RP = 25 (UFRN)	NA	NA	NA	NA	NA
NPS, sample 3	2020 Oct 11	2020 Oct 13	Positive, C_t_ E = 25, C_t_ RP = 24 (LACEN-PB)	Positive, C_t_ E = 22, C_t_ N1 = 23, C_t_ N2 = 22, C_t_ RP = 26	GR/P.2	Positive	NA	NA
Serum specimen, sample 4	NA	2020 Dec 13	NA	NA	NA	NA	Positive	B.1<10, P.1<10, P.2<10, B.1.1.7<10

*CPE, cytopathic effect; C_t_, cycle threshold; E, envelope protein; LACEN-PB, Laboratório Central do Estado da Paraíba; LVRS, Respiratory Virus and Measles Laboratory; N1 and N2, portion of nucleoprotein; NA, not applicable; ND, not detected; NPS, nasopharyngeal swab specimen; PANGO, Phylogenetic Assignment of Named Global Outbreak Lineages; RP, human RNase P gene; RT-PCR, reverse transcription PCR; SARS-CoV-2, severe acute respiratory syndrome coronavirus 2; UFRN, Federal University of Rio Grande do Norte.

This case was confirmed as a reinfection according to the Ministry of Health of Brazil criteria for reinfection confirmation, which stipulates 2 positive rRT-PCR results separated by >90 days. The 2 positive samples were collected 116 days apart. Viral isolation from the specimen collected in the second episode of COVID-19 was negative for infectious virus in VERO E6 cells culture after 2 passages.

Serum sample collected 2 months after the second episode tested positive for IgG by CMIA, which uses the nucleocapsid protein of SARS-CoV-2. However, when the same serum sample was tested by PRNT for B.1, P.1, P.2, and B.1.1.7 lineages, neutralizing antibodies were under the detectable level of our assay; PRNT_90_ titers for all 4 lineages were <10.

### Genomic Findings

**Figure 1:**
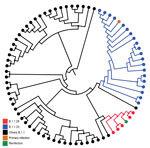
Maximum-likelihood tree of severe acute respiratory syndrome coronavirus 2 whole-genome sequences from Paraíba in study of reinfection case, Brazil, June–October 2020. Branching pattern of whole-genome sequences (29779 nt) from Paraíba (n = 77) are shown classified within lineages B.1.1.28 (red), B1.1.33 (blue), and others B.1.1 (black). Sequences derived from the primary infection and reinfection are highlighted with different colors as indicated. Nodes with high statistical support (approximate-likelihood ratio test >9.0) are marked by the smaller circular shapes.

To distinguish between reinfection and long-term viral persistence, we recovered the SARS-CoV-2 whole genomes from the 2 positive nasopharyngeal swab specimens (accession nos. EPI_ISL_792561 and EPI_ISL_792562) of the reinfection case plus 76 SARS-CoV-2 positive cases detected in the same state of Paraíba during April 6–November 27, 2020 (EPI_ISL_792563 to EPI_ISL_792638). We performed maximum-likelihood phylogenetic analysis of all SARS-CoV-2 whole genomes from the state of Paraíba by using PhyML (*8*); this analysis revealed 2 different viral lineages in the 2 COVID-19 episodes. In the first episode, we detected the lineage B.1.1.33, whereas lineage P.2 (alias for B.1.1.28.2) was detected in the third clinical specimen (from the second episode) (Figure 1), according to PANGO lineage classification (*10*). The SARS-CoV-2 B.1.1.33 lineage was also detected in other samples from the state of Paraíba (Figure 1) and represent the most prevalent viral variant circulating in Brazil during the early epidemic phase (*11*,*12*). Of note, sequences recovered from the reinfection case and from 2 additional cases in the state of Paraíba harbor the substitution S-E484K (G23012A) and were classified as lineage P.2, which was initially detected in the state of Rio de Janeiro (*13*).

**Figure 2:**
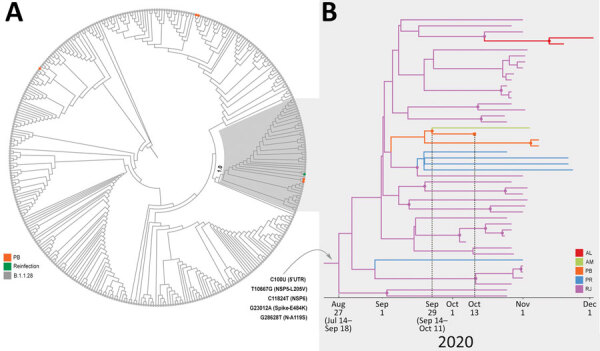
Emergence of the P.2 clade in study of severe acute respiratory syndrome coronavirus 2 (SARS-CoV-2) reinfection case, Brazil. A) Maximum-likelihood phylogenetic tree of B.1.1.28 SARS-CoV-2 whole-genome sequences (29,779-nt) from Brazil (n = 376). Shaded box highlights the P.2 clade (n = 47), and its statistical support (approximate-likelihood ratio test = 1.0) is indicated in the cladogram. Sequences from Paraíba are indicated in orange and sequences from the reinfection case are indicated by green. B) Time-scaled Bayesian maximum clade credibility tree of SARS-CoV-2 whole-genome sequences from the P.2 clade (n = 47). Branches are colored according to the most probable location state of their descendent nodes as indicated. The 5 lineage-defining single-nucleotide polymorphisms are indicated at the maximum clade credibility tree root node. Circular shapes mark nodes with high statistical support (posterior probability>9.0), and a square tip shape indicates the sequence from reinfection case. AL, Alagoas; AM, Amazonas; PB, Paraíba; PR, Parana; RJ, Rio de Janeiro; UTR, untranslated region.

To better characterize the P.2 virus detected in the second SARS-CoV-2–positive nasopharyngeal swab specimen, we aligned it against all B.1.1.28 (an ancestor of P.2) whole genomes available in the GISAID EpiCoV database as of December 20, 2020. In addition, we also selected 8 P.2 whole-genome sequences from the states of Alagoas (n = 2), Amazonas (n = 1), and Parana (n = 5) available in the Fiocruz COVID-19 Genomic Surveillance Network database (accession nos. EPI_ISL_792560, EPI_ISL_792639, EPI_ISL_792642, EPI_ISL_792645, EPI_ISL_792646, and EPI_ISL_792650–52). The new maximum-likelihood phylogenetic tree revealed that the lineage P.2 recovered from the reinfection case branched in a highly supported (approximate-likelihood ratio test = 1) subclade with 46 additional sequences sampled during October–December, 2020, in the states of Rio de Janeiro, Paraíba, Alagoas, Parana, and Amazonas (Figure 2, panel A). We identified 5 lineage-defining single-nucleotide polymorphisms: C100U (5′ untranslated region), T10667G (NSP5_L205V), C11824T (NSP6), G23012A (S_E484K), and G28628T (N_A119S) that distinguish P.2 sequences from all other B.1.1.28 sequences available in Brazil.

To further investigate the spatiotemporal emergence of the VOI P.2 and the viral strain detected in the reinfection case, we conducted a Bayesian phylogeographic analysis of all 47 SARS-CoV-2 sequences in Brazil that branched within such clade. We estimated time-scaled trees by using a strict molecular clock model with a uniform substitution rate prior (8–10 × 10^−4^ substitutions/site/year), a general time-reversible plus invariable sites plus gamma nucleotide substitution model, and the Bayesian skyline coalescent prior as implemented in BEAST version 1.10 (*9*). Bayesian reconstructions traced the origin of the lineage P.2 in the state of Rio de Janeiro (PSP = 0.97) on August 27 (95% highest posterior density credible interval July 14–September 18) and its subsequent dispersion from Rio de Janeiro to other states in the southern and northeastern regions of the country (Figure 2, panel B). This phylogeographic reconstruction also supports a dissemination event from the state of Paraíba to the state of Amazonas and the branching of the P.2 sequence from the reinfection case with that from Amazonas with high support (PP = 1) (Figure 2, panel B). The most recent common ancestor of P.2 sequences from the reinfection case and the state of Amazonas was dated to September 29 (95% highest posterior density credible interval September 14–October 11), a few days before the onset of reinfection symptoms on October 11.

## Discussion

We demonstrate that this reinfection case in Brazil corresponds to a primary infection with the lineage B.1.1.33 and a reinfection with the VOI P.2, which harbors the mutation S-E484K. The age of the common ancestor of the P.2 virus of the reinfection case and a nonrelated virus sampled in the state of Amazonas provide a maximum limit for the reinfection episode during September 14–October 11. The estimated period excludes the possibility of long-term persistence of the P.2 virus since primary infection (before June 23, 2020).

Of note, the reinfection case reported here coincides with a recently reported case in the state of Bahia that also described a primary infection with the B.1.1.33 variant and reinfection with the P.2 viral variants (*14*). These studies also confirm that the P.2 initially described in the state of Rio de Janeiro (*13*) is more widely distributed across different states in Brazil. Our analysis supports that the P.2 lineage probably emerged in Rio de Janeiro around late August, but defining the precise location and time of emergence of this novel lineage will require a denser sampling from different states in Brazil from the second half of 2020.

The mutation E484K is located in the receptor-binding domain and has also been recently described in multiple SARS-CoV-2 VOI and variants of concern rapidly spreading in the Americas, Europe, and Africa (*15*). The rapid dissemination of these variants, combined with the ability of viruses harboring the mutation E484K to potentially escape from neutralizing antibodies mounted for older lineages (*13*,*16*), should raise concern about the potential effect on infectivity, pathogenicity, and reinfection.

We also speculate that the reinfection case described resulted from a weak and transient protective immunity that occurred after primary infection. Consistent with this notion, despite the positive result for IgG by CMIA in the serum sample collected 2 months after the second SARS-CoV-2 infection, PRNT_90_ titers for all 4 lineages of SARS-CoV-2 tested, including P.2, were below the detectable level. The prevalence of neutralizing antibodies also varies among patients and low levels or absence of neutralizing antibody has been reported in mildly affected COVID-19 convalescent patients (*17*). In a study conducted with SARS-CoV-2–infected healthcare workers, neutralizing activity rapidly declined and might even be lost beginning 2 months after disease onset (*18*).

Whether reinfected persons might contribute substantially to the onwards transmission of SARS-CoV-2 in the population is currently unclear. The negative results from viral isolation after 2 sequential passages of nasopharyngeal swab specimens suggests absence (or low levels) of infectious virus in the second episode of COVID-19. Viral isolation prevalence among COVID-19 patient samples varies and is usually lower in mild infections with high C_t_ values (*17*).

Our results demonstrate that previous exposure to SARS-CoV-2 might not guarantee immunity, and that sequential infections might not mount detectable neutralizing antibodies in all cases. These findings reinforce the need to maintain nonpharmacologic protective measures not only by persons who test negative but also for those who have already tested positive for SARS-CoV-2. Characterization of the immune response in persons who become reinfected with SARS-CoV-2 will be crucial to learn more about the role of viral and host factors on this rare phenotype.

Appendix Additional data used in study of severe acute respiratory syndrome coronavirus 2 P.2 lineage associated with reinfection case, Brazil, June–October 2020

## References

[1] Babiker A, Marvil CE, Waggoner JJ, Collins MH, Piantadosi A. The importance and challenges of identifying SARS-CoV-2 reinfections. J Clin Microbiol. 2021;59:e02769–20. 33361342 10.1128/JCM.02769-20 PMC8092746

[2] Brazilian Ministry of Health, Health Surveillance Secretariat. TECHNICAL NOTE Nº 52/2020-CGPNI/DEIDT/SVS/MS [in Portuguese]. In: Department of Immunization and Communicable Diseases. General Coordination of the National Immunization Program, editors. Bairro Asa Norte: Brazil DF; 2020. p. 4.

[3] National Institutes of Health. Clinical spectrum of SARS-CoV-2 infection. December 17, 2020 [cited 2021 Mar 29]. https://www.covid19treatmentguidelines.nih.gov/overview/clinical-spectrum

[4] Biomanginhos FIOCRUZ. Kit Molecular SARS-CoV-2 (information and consultation of manuals) [in Portuguese] [cited 2020 Apr 15]. https://www.bio.fiocruz.br/index.php/br/produtos/reativos/testes-moleculares/novo-coronavirus-sars-cov2

[5] Food and Drug Administration. CDC 2019-novel coronavirus (2019-nCoV) real-time RT-PCR diagnostic panel [cited 2020 Dec 1]. https://www.fda.gov/media/134922/download 10.1371/journal.pone.0260487 PMC8673615 34910739

[6] Basile K, McPhie K, Carter I, Alderson S, Rahman H, Donovan L, et al. Cell-based culture of SARS-CoV-2 informs infectivity and safe de-isolation assessments during COVID-19. Clin Infect Dis. 2020;ciaa1579; Epub ahead of print. 10.1093/cid/ciaa1579 33098412 PMC7665383

[7] Deshpande GR, Sapkal GN, Tilekar BN, Yadav PD, Gurav Y, Gaikwad S, et al. Neutralizing antibody responses to SARS-CoV-2 in COVID-19 patients. Indian J Med Res. 2020;152:82–7. 10.4103/ijmr.IJMR_2382_20 32859866 PMC7853248

[8] Guindon S, Dufayard JF, Lefort V, Anisimova M, Hordijk W, Gascuel O. New algorithms and methods to estimate maximum-likelihood phylogenies: assessing the performance of PhyML 3.0. Syst Biol. 2010;59:307–21. 10.1093/sysbio/syq010 20525638

[9] Suchard MA, Lemey P, Baele G, Ayres DL, Drummond AJ, Rambaut A. Bayesian phylogenetic and phylodynamic data integration using BEAST 1.10. Virus Evol. 2018;4:vey016. 10.1093/ve/vey016 29942656 PMC6007674

[10] Rambaut A, Holmes EC, O’Toole Á, Hill V, McCrone JT, Ruis C, et al. A dynamic nomenclature proposal for SARS-CoV-2 lineages to assist genomic epidemiology. Nat Microbiol. 2020;5:1403–7. 10.1038/s41564-020-0770-5 32669681 PMC7610519

[11] Candido DS, Claro IM, de Jesus JG, Souza WM, Moreira FRR, Dellicour S, et al.; Brazil-UK Centre for Arbovirus Discovery, Diagnosis, Genomics and Epidemiology (CADDE) Genomic Network. Evolution and epidemic spread of SARS-CoV-2 in Brazil. Science. 2020;369:1255–60. 10.1126/science.abd2161 32703910 PMC7402630

[12] Resende PC, Delatorre E, Gräf T, Mir D, Motta FC, Appolinario L, et al. Evolutionary dynamics and dissemination pattern of the SARS-CoV-2 lineage B.1.1.33 during the early pandemic phase in Brazil. Front Microbiol. 2020; [Epub ahead of print]. 33679622 10.3389/fmicb.2020.615280 PMC7925893

[13] Voloch CM, da Silva Francisco R Jr, de Almeida LGP, Cardoso CC, Brustolini OJ, Gerber AL, et al. Covid19-UFRJ Workgroup, LNCC Workgroup, Adriana Cony Cavalcanti. Genomic characterization of a novel SARS-CoV-2 lineage from Rio de Janeiro, Brazil. J Virol. 2021;JVI.00119-21; [Epub ahead of print]. 10.1128/JVI.00119-21 PMC8139668 33649194

[14] Nonaka CKV, Franco MM, Gräf T, de Lorenzo Barcia CA, de Ávila Mendonça RN, de Sousa KAF, et al. Genomic evidence of SARS-CoV-2 reinfection involving E484K spike mutation, Brazil. Emerg Infect Dis. 2021 Feb 19 [Epub ahead of print]. 10.3201/eid2705.210191 PMC8084516 33605869

[15] Latif AA, Mullen JL, Alkuzweny M, Tsueng G, Cano M, Haag E, et al. S:E484K mutation report. 2020 [cited 2021 Mar 25]. https://outbreak.info/situation-reports?muts=S%3AE484K

[16] Liu Z, VanBlargan LA, Bloyet L-M, Rothlauf PW, Chen RE, Stumpf S, et al. Identification of SARS-CoV-2 spike mutations that attenuate monoclonal and serum antibody neutralization. Cell Host Microbe. 2021;29:477–488.e4. 10.1016/j.chom.2021.01.014 33535027 PMC7839837

[17] Walsh KA, Jordan K, Clyne B, Rohde D, Drummond L, Byrne P, et al. SARS-CoV-2 detection, viral load and infectivity over the course of an infection. J Infect. 2020;81:357–71. 10.1016/j.jinf.2020.06.067 32615199 PMC7323671

[18] Marot S, Malet I, Leducq V, Zafilaza K, Sterlin D, Planas D, et al.; Sorbonne Université SARS-CoV-2 Neutralizing Antibodies Study Group. Rapid decline of neutralizing antibodies against SARS-CoV-2 among infected healthcare workers. Nat Commun. 2021;12:844. 10.1038/s41467-021-21111-9 33558507 PMC7870823

